# Biotinylation Interferes with Protein Ubiquitylation and Turnover in Arabidopsis—A Cautionary Insight for Proximity Labeling in Ubiquitylation Proteome Studies

**DOI:** 10.3390/ijms26178248

**Published:** 2025-08-25

**Authors:** Yang Li, Peifeng Yu, Zhihua Hua

**Affiliations:** 1 Department of Environmental and Plant Biology, Ohio University, Athens, OH 45701, USA; yl668319@ohio.edu (Y.L.); py989117@ohio.edu (P.Y.); 2 Interdisciplinary Program in Molecular and Cellular Biology, Ohio University, Athens, OH 45701, USA

**Keywords:** TurboID, cullin-RING E3 ligases, SCF, phytochrome A, biotinylation, ubiquitylation

## Abstract

Nearly all eukaryotic proteins are turned over by the ubiquitin (Ub)-26S proteasome system (UPS). Despite its broad cellular roles, only a handful of UPS members, particularly the Ub E3 ligases that specifically recognize a protein for ubiquitylation, have been characterized in plants to date. The challenge arises from the transient recognition and rapid degradation of ubiquitylation substrates by the UPS. To tackle this challenge, the emerging biotinylation-based proximity labeling (PL) offers an exciting tool for enriching transient interactors of Ub E3 ligases. In this study, we examined the efficacy of TurboID in identifying substrates of Arabidopsis Skp1-cullin1-F-box (SCF) ligases. We demonstrate that the Arabidopsis Skp1 Like (ASK)1-TurboID is not fully functioning in planta, which led us to discover a novel antagonism between biotinylation and ubiquitylation in regulating protein stability in vivo. This discovery lowers the effectiveness of PL in ubiquitylome studies. However, using one long-known SCF substrate, phytochrome A, we succeeded to apply its TurboID fusion for complementing the far-red-light response of the *phyA-211* null mutant allele, suggesting an efficacy of PL in characterizing single ubiquitylation pathways. This study highlighted a limitation of PL in ubiquitylome studies, discovered a new antagonistic pathway of biotinylation, and developed a theoretical guidance for future PL-based characterization of ubiquitylation pathways.

## 1. Introduction

The ubiquitin (Ub)-26S proteasome system (UPS) constitutes the largest functional protein community in the plant proteome [1]. In the model flowering plant *Arabidopsis thaliana* (Arabidopsis hereafter), nearly 6% of its annotated protein-coding genes encode UPS components [2,3]. The UPS operates as two tandem subsystems: the ubiquitylation machinery, which selectively tags obsolete or aberrant proteins with Ub, and the 26S proteasome complex, which recognizes and degrades the ubiquitylated proteins. The ubiquitylation process is executed by a cascade of three enzymes: (i) E1, which activates Ub in an ATP-dependent reaction; (ii) E2, which conjugates with an activated Ub to form a high-energy Ub-E2 thioester; and (iii) E3, which recruits the substrate and positions its lysine residue in the Ub-E2 active center, catalyzing the formation of an isopeptide bond with the C-terminal Gly of Ub [4].

Although the molecular choreography between E2 and E3 during Ub-chain assembly remains incompletely understood, substrate specificity is primarily conferred by the extensive E3 ligase family [5]. In plants, the largest E3 class consists of multi-subunit cullin (CUL)-RING ligases (CRLs), each built from a conserved CUL-RBX catalytic core and interchangeable substrate-receptor modules (SRMs) [6]. Given the specificity of SRMs and the CUL proteins, three major CRL types are present in Arabidopsis that include CUL1^F-box^ complexes (also known as Skp1-CUL1-F-box, SCF), CUL3a/3b^BTB^ complexes, and CUL4^DDB^ complexes [7]. A handful of characterized CRL complexes have demonstrated their myriad functions in plants from serving as receptors of hormones and light, participating in signaling transduction, to controlling the cell cycle, transcription activity, stress response, self-incompatibility, and pathogen defense [7]. Unfortunately, systematic dissection of these pathways is hampered by the low expression of many E3 genes, the sheer size of the E3 repertoire, rapid turnover of ubiquitylated substrates, and the transient nature of E3–substrate interactions. To overcome these challenges, we explored biotin-based proximity labelling (PL), which captures weak and transient interactors through the strong biotin–streptavidin affinity [8].

Biotin-based PL employs genetically engineered promiscuous biotin ligases (PBLs) or ascorbate peroxidase (APEX) for generating reactive biotin derivatives that diffuse ~10–20 nm from the enzyme’s active center and covalently tag nearby proteins [9,10,11]. Biotinylated proteins are then purified stringently via streptavidin affinity, enabling the recovery of weak or transient E3-substrate interactomes. Owing to the requirement of toxic hydrogen peroxide and high endogenous peroxidase activity in plant cells, APEX has seen limited plant use. Instead, plant studies rely on PBLs.

Since the discovery of the first PBL, successive PBL generations, including BioID (i.e., BirA*) [12], BioID2 [13], TurboID and mini-Turbo [14], and UltraID [15], have progressively improved labelling efficiency. Early plant BioID studies identified only limited proximal interactomes [16,17,18,19], partly because BioID/BioID2 labelling conditions are sub-optimal in planta [20]. TurboID, which achieves rapid and non-toxic labeling in as little as 10 min, markedly expanded plant PL. For example, through the stable expression of a TurboID-fused FAMA transcription factor in Arabidopsis guard cells, 47 high-confidence FAMA interactors were identified [21]. Similarly, TurboID has also profiled proteins proximal to the N immune receptor in *Nicotiana benthamiana* [22], mapped protein–protein interactions across multiple plant systems [20], characterized meiotic chromosome axes [23], and delineated the phosphoproteome associated with Arabidopsis BRASSINOSTEROID-INSENSITIVE 2 [24].

The effective PL of TurboID in plant cells makes it possible to illuminate E3-substrate regulatory proteomes. Indeed, BioID-based PL was used to recover 77 candidate substrates of the F-box proteins, β-transducin repeat-containing protein (β-TrCP) 1 and 2, in human cells [25]. In plants, a transient TurboID expression system in Arabidopsis rosette leaves was also explored to identify 13 F-box-containing SRMs using TurboID-fused Arabidopsis Skp1-Like 1 (TurboID-ASK1) or TurboID-fused Arabidopsis CUL1 N-terminal domain (TurboID-CUL1^NTD^) [8]. However, the number of F-box proteins identified is far below expectations, given that there are over 800 annotated *F-box* genes and 129 *Core Arabidopsis F-box* (*CAF*) genes [2,26,27]. To resolve this discrepancy, we generated stable TurboID lines and, through careful evaluation, unexpectedly uncovered a novel antagonism between biotinylation and ubiquitylation pathways in plants.

## 2. Results

### 2.1. Generating ASK1-TurboID Transgenic Plants

**Figure 1:**
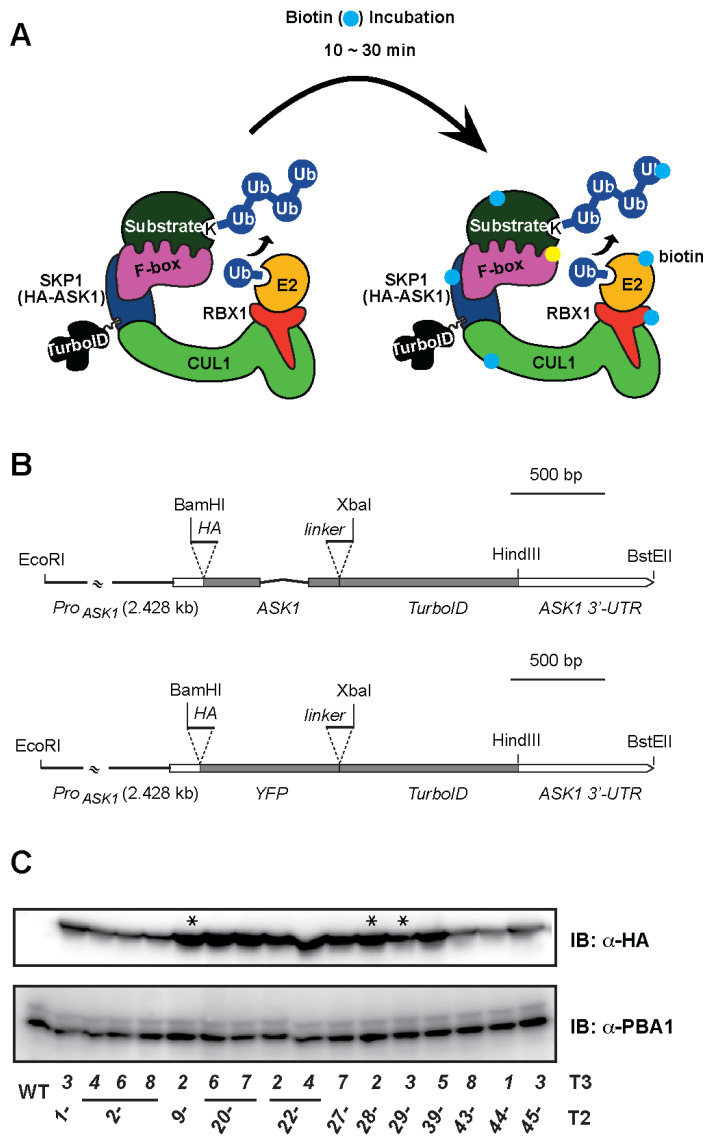
Raising homozygous *HAT* transgenic plants. (**A**) A schematic diagram showing the structural composition of an HAT-containing SCF complex and putative biotin-labeling on proteins in proximity to HAT. (**B**) Structural diagrams showing the DNA fragments and restriction sites used for *HAT* (**top panel**) and *HYT* (**bottom panel**) constructions. (**C**) Immunoblot analysis with anti-HA antibody reveals varying levels of HAT products in 12 independent *HAT* homozygous transgenic lines, likely resulting from transgene position effects. PBA1 was used as a loading control to verify comparable protein input. Asterisks indicate the three *HAT* lines selected for the complementation assay.

To profile ubiquitylation pathways in vivo, it is essential to introduce a PBL fusion protein with preserved biological function because mis-regulation of an E3 ligase can disrupt the proteome homeostasis. To reach this goal, we sought to study the SCF-mediated ubiquitylation system for two reasons. First, the SCFs are the founding members of CRLs, comprising a Skp1-F-box SRM and a CUL1-RBX1 catalytic core [6]. In this complex, the F-box recruits SCF substrates, while Skp1 acts as a molecular bridge linking the F-box-substrate to the CUL1-RBX1 scaffold. Therefore, Skp1 is an ideal bait to label proximal interactors of active SCF complexes and their F-box components (Figure 1A). Second, among the 21 Skp1-like proteins in Arabidopsis, ASK1 has been demonstrated to be the predominant member [28,29,30]. In a previous study, we had generated a *Ds*-insertion *ask1* null mutant in the Col-0 background [31]. The *ask1* mutant has a striking low-fertility phenotype with abnormal floral structures, making it a suitable background to test the complementation function of a TurboID-fused ASK1 protein.

To closely represent the endogenous tissue-specific transcriptional activity of *ASK1*, we cloned a 2.4 kb genomic sequence upstream of the *ASK1* start codon as the promoter to drive the expression of the transgene. We also utilized the genomic region that covers the open reading frame (ORF) of *ASK1* to fuse in-frame with the 5′-end of *TurboID* coding sequence. To benefit immunoblotting detection, the coding sequence for an HA epitope tag was inserted in-frame at the 5′-end of *ASK1* ORF. Additionally, a 735-bp 3′ untranslated region (UTR) downstream of the *ASK1* stop codon was cloned and fused to the 3′ end of *TurboID*. The resulting *Pro_ASK1_:HA-ASK1-TurboID:ASK1-3′-UTR* fragment was ligated into a binary vector pCAMBIA1302 to create the *HA-ASK1-TurboID* (*HAT*) construct. As a control, the *ASK1* ORF was replaced with the coding sequence of Yellow Fluorescent Protein (YFP) to yield the *HA-YFP-TurboID* (*HYT*) construct (Figure 1B).

**Figure 2:**
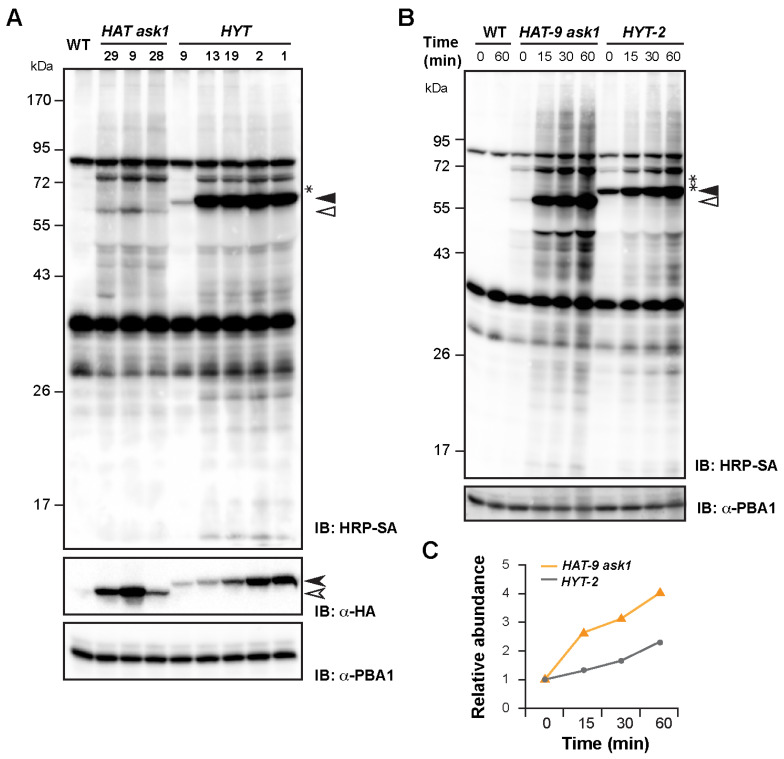
HAT does not yield many specific trans-biotinylation species that are immuno-detectable. (**A**) *HAT ask1* and *HYT* share a similar background profile of detectable trans-biotinylation. Total protein of 7 d-old LD-grown seedlings of the indicated independent transgenic lines and WT was subjected to immunoblot analysis. HRP-SA and anti-HA antibody were used to detect biotinylated proteins and transgene products, respectively. Open and solid triangles indicate cis-biotinylated HAT and HYT, respectively. Open and solid arrowheads indicate the corresponding total HAT and HYT protein products, respectively. The single HAT-specific trans-biotinylation species is shown by an asterisk. PBA1 served as a loading control, as described in Figure 1C. (**B**) Immunoblotting analysis demonstrates a more rapid increase in both cis- and trans-biotinylation in *HAT-9 ask1* compared to *HYT* seedlings over a time course of incubation with 50 μM biotin. Open and solid triangles mark cis-biotinylated HAT and HYT, respectively. The asterisk denotes the HAT-specific trans-biotinylation species. PBA1 served as a loading control, as in (**A**). (**C**) Densitometric quantification of total biotinylated proteins confirms a higher rate of biotinylation in *HAT-9 ask1* than that of *HYT*. Band intensities were normalized to the 0 min background biotinylation level for each genotype.

Through transformation, we obtained 12 T3 homozygous lines that carry one single-copy *HAT* transgene insertion (Figure 1C). To determine an optimal expression level of HAT for specific PL species production, we selected three independent transformants, *HAT9*, *HAT28*, and *HAT29*, representing high, intermediate, and low HAT production, respectively, based on immunoblotting analysis against HA (Figure 1C and Figure 2A). These lines were crossed into the *ask1* mutant to generate *HAT ask1* double homozygous plants for complementation analysis. In parallel, we also developed five T4 homozygous lines carrying one single-copy *HYT* insertion in the Col-0 background. Like *HAT* plants, the *HYT* lines also showed varying HYT protein levels (Figure 2A), which allowed us to compare biotinylation specificity between HAT and HYT while minimizing differences due to expression dosage.

### 2.2. The HAT-Specific Proximal Interactome Is Shaded by Nonspecific PL

Since plant cells synthesize biotins endogenously [32], we hypothesized that PL could already be active in *HAT ask1* and *HYT* plants without exogenous biotin supplementation. To test this, we analyzed 7-day(d)-old seedlings of *HAT ask1* and *HYT* lines by immunoblotting using horseradish-peroxidase-conjugated streptavidin (HRP-SA), which detects biotinylated proteins. A clear PBL dosage-dependent increase in biotinylated species was observed in both *HAT ask1* and *HYT* plants (Figure 2A). Both lines showed strong cis-biotinylation (i.e., self-biotinylation of HAT and HYT), as well as multiple discrete trans-biotinylation bands that were absent in WT controls.

However, only one band appeared to be unique to *HAT* (Figure 2A, single asterisk), suggesting that the majority of trans-biotinylation signals are nonspecific. Given ASK1′s role in assembling a large group of SCF complexes, the *HAT* plants were expected to yield more distinct trans-biotinylation products than the *HYT* plants (Figure 1A). The limited HAT-specific signal implies the need for PL condition optimization. One possible explanation is that the ASK1-TurboID fusion in *HAT* is less accessible to endogenous biotin due to conformational constraints or SCF complex formation. Supporting this, although *HAT-28 and HAT-9* showed higher protein levels of TurboID fusions than *HYT-13 and HYT-19* as detected by anti-HA antibody, their cis-biotinylation levels were dramatically lower (Figure 2A), reinforcing the hypothesis that biotin availability or accessibility may be limiting.

To further explore the effect of biotin availability, we conducted a time-course PL assay using *HAT-9 ask1* and *HYT-2*, which express comparable levels of TurboID fusion proteins (Figure 2A). Seedlings were incubated in 50 µM exogenous biotin for various time periods up to 60 min (Figure 2B). Consistent with our hypothesis, *HAT-9 ask1* showed a rapid increase in both cis- and trans-biotinylation upon biotin treatment, exceeding the biotinylation rate of *HYT-2* (Figure 2B,C). However, despite this increase, only a few trans-biotinylation bands (possibly two) appeared to be specific to *HAT-9 ask1* throughout the time course (Figure 2B), suggesting that nonspecific PL signals persist and may obscure detection of specific ASK1-associated interactors.

These nonspecific products likely obscure detection of specific biotinylation events involving low-abundance proteins, such as F-box members in SCF complexes. The biotinylated F-box proteins may be present but remain undetectable by standard immunoblotting. The high background of nonspecific trans-biotinylation products could also interfere with downstream streptavidin affinity purification (AP) and compromise the identification of ASK1-specific interactors by mass spectrometry (MS) analysis.

### 2.3. HAT Partially Complements Ask1′s Growth and Reproductive Defects

**Figure 3:**
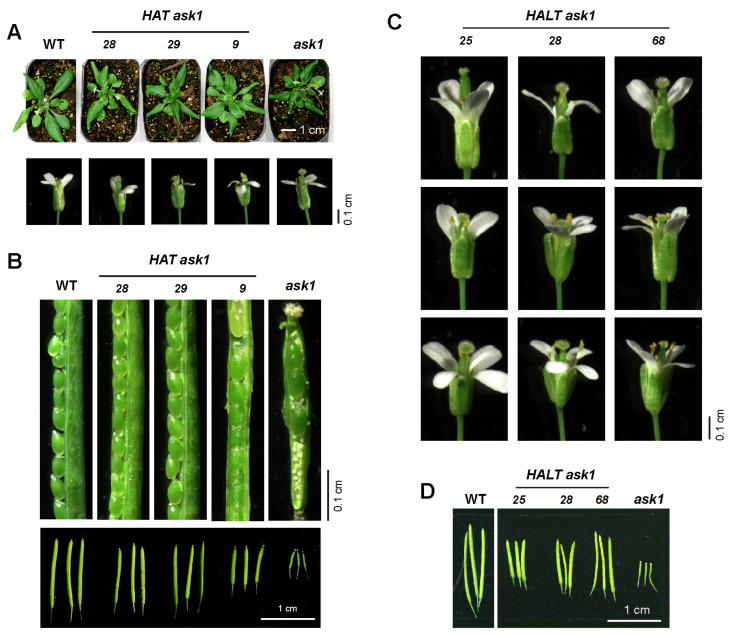
Partial complementation activities of HAT and HALT in rescuing *ask1*′s growth and reproductive defects. (**A**) Twisted rosette leaves and floral organs with fewer than four floral petals indicate incomplete complementation in *HAT ask1* plants. Scale bars are provided throughout the manuscript to indicate the size of tissues or organs being compared. (**B**) Expression of *HAT* partially rescues seed fertilization and silique development in *HAT ask1* plants, as evidenced by increased seed set and enlarged siliques. (**C**,**D**) Introducing a long linker between ASK1 and TurboID does not improve the partial complementation activity of HAT in floral (**C**) and silique (**D**) development in *HALT ask1* plants.

A straightforward genetic test for functional fidelity of the HAT fusion is its ability to rescue the characteristic *ask1* defects, including twisted rosette leaves, aberrant floral organs with variable petal numbers, markedly reduced fertility, and short siliques [31]. Developmental analyses revealed that all three *HAT ask1* lines retained twisted rosette leaves and abnormal flowers, although their fertility and silique length were restored to varying degrees (Figure 3A,B). Notably, the extent of rescue appeared to be inversely correlated with HAT protein abundance. For example, *HAT9 ask1*, which expressed the highest level of HAT (Figure 2A), displayed the least recovery of seed set and silique length among the three lines (Figure 3B).

The partial complementation suggested that the TurboID moiety might sterically impair ASK1 function. Because the original HAT fusion contained an 8-amino-acid linker (AGGGGPSR) between ASK1 and TurboID (Figure 1B), we replaced it with a 21-amino acid linker (AGGGGPSRGAAGGGGGGGGGG) to increase the flexibility of TurboID [20]. We designated the new fusion gene as *HA-ASK1-Long-linker-TurboID* (*HALT*). However, upon introducing *HALT* in three new independent *ask1* complemented lines, it did not improve the reproductive traits compared with the corresponding *HAT* lines, including shortened stamens, reduced petal numbers, and only partial recovery of silique length (Figure 3C,D). Hence, the incomplete complementation is unlikely to result solely from linker length or fusion architecture, implying that an inherent functional interference, possibly the biotinylation activity itself, limits full rescue.

### 2.4. Antagonism Between Biotinylation and Ubiquitylation Prevents a Normal Function of HAT

**Figure 4:**
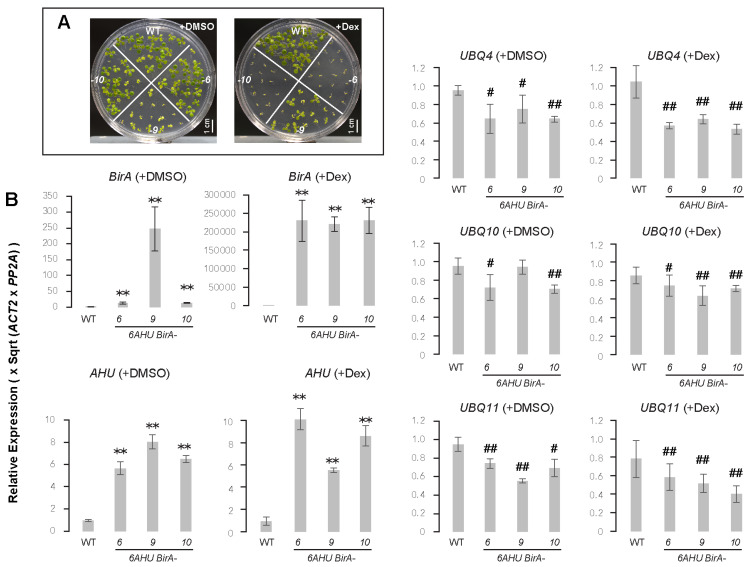
Expression of transgenes and endogenous *UBQ* genes in response to Dex treatment in *6AHU BirA* homozygous lines. (**A**) Severe growth inhibition of 12-d-old *6AHU BirA* seedlings compared to WT grown on 1/2 MS medium supplemented with either DMSO (0.1% *w*/*v*) or 10 μM Dex. The pronounced growth defect of *6AHU BirA-9* treated with DMSO is attributed to its higher basal *BirA* expression compared to other lines as indicated in (**B**). (**B**) qPCR analysis of *BirA*, *AHU*, *UBQ4*, *UBQ10*, and *UBQ11* transcript levels. Expression values were normalized to one WT biological replicate under the same treatment (DMSO or Dex). Bars represent mean ± SD from three biological replicates, each with three technical replicates. Asterisks and hash symbols indicate statistically significant upregulation and downregulation, respectively (Student’s *t*-test, # *p* < 0.05, ** and ## *p* < 0.01).

**Figure 5:**
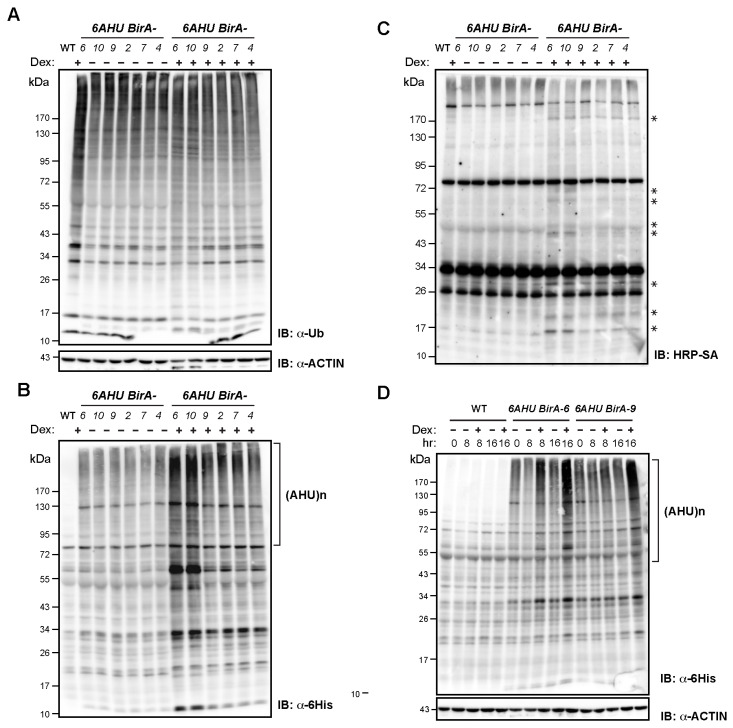
Biotinylation interferes with normal ubiquitylation function by stabilizing ubiquitylated proteins. Immunoblot analysis of *6AHU BirA* homozygous lines following Dex-induced *BirA* expression. Dex-treated WT seedlings served as positive controls for total ubiquitylated proteins and negative controls for AHU-conjugated species. Sample numbers indicate independent *6AHU BirA* homozygous lines. Total protein was extracted from 7 d-old seedlings grown on 1/2 MS medium, followed by either a 16 h treatment (**A**–**C**) or the indicated time periods (**D**) in liquid 1/2 MS medium containing 50 μM biotin and either 0.1% DMSO (–) or 10 µM Dex (+). Proteins were resolved by 6–20% gradient SDS-PAGE and blotted with the indicated antibodies. Brackets mark high-molecular-mass AHU-conjugated species. ACTIN was used as a loading control. (**A**) Total ubiquitylated proteins are reduced in *6AHU BirA* lines under basal conditions and partially restored upon Dex treatment. (**B**) Dex treatment stabilizes AHU-conjugated proteins. (**C**) Dex treatment enhances biotinylation of ubiquitylated proteins, as indicated by asterisks. (**D**) Time-course analysis showing that Dex, but not DMSO, progressively increases AHU-conjugated proteins in *6AHU BirA-6* and *-9*.

Since a basal level of biotinylation is present in *HAT* seedlings (Figure 2A), we asked whether protein biotinylation might antagonize ubiquitylation. To test this idea, we generated double transgenic plants carrying (i) a recombinant *polyubiquitin* (*UBQ*) gene comprising six *AviTag-6His*-tagged *Ub* moieties (designated *6AHU*), and (ii) the *Escherichia coli* biotin ligase gene *BirA* (Figure S1A,B). Our initial experiments showed that constitutive co-expression of both transgenes was unattainable, as no transformants stably harboring both were recovered. This result implied that high levels of biotinylated Ub are developmentally lethal. To overcome this limitation, we placed *6AHU* under the control of the endogenous *UBQ10* promoter and *BirA* behind a dexamethasone (Dex)-inducible promoter to minimize background *BirA* expression and, consequently, basal production of biotinylated Ub (Figure S1A,B). We sequentially transformed *6AHU* and *BirA* into Col-0 to obtain six independent *6AHU BirA* homozygous lines that share the same *6AHU* insertion but differ in *BirA* loci, allowing us to examine how varying degrees of biotinylation affect the functions of ubiquitylation substrates (Figure 4, Figure 5 and Figure S1).

#### 2.4.1. Mild Reduction of Endogenous *UBQ* Transcripts Reduces Total Ubiquitylated Proteins

After growing the transgenic and WT seedlings on half-strength Murashige–Skoog (1/2 MS) medium supplemented with DMSO (0.1% *w*/*v*) or 10 μM Dex for 12 days under a long-day (LD) photoperiod (Figure 4A), we analyzed relative *BirA* expression using qPCR in three selected *6AHU BirA* lines. Under a DMSO mock treatment, *BirA* expression increased by 13 ± 3, 247 ± 70, and 13 ± 1.8 fold in *6AHU BirA-6*, *-9*, and *-10*, respectively (mean ± SD, and hereafter), in comparison with WT grown under the same conditions. Upon Dex treatment, *BirA* expression in these three lines increased dramatically to 2.3 ± 0.6 × 10^5^, 2.2 ± 0.2 × 10^5^, and 2.3 ± 0.3 × 10^5^ fold, respectively, compared to WT (Figure 4B). These results indicate effective inducible expression of *BirA* in all three transgenic lines.

To investigate how the abundance of total ubiquitylated protein changed in response to Dex-induced *BirA* expression, we performed immunoblotting assays using anti-Ub antibodies. We observed reduced total ubiquitylated proteins in six *6AHU BirA* transformants, including those of three described above, under DMSO mock treatment compared to WT (Figure 5A). Since *BirA* expression was minimal without Dex, particularly in *6AHU BirA-6*, and -*10* (Figure 4B), the reduced levels of total ubiquitylated proteins suggest that basal BirA-mediated biotinylation of Ub may interfere with normal ubiquitylation, or that *AHU* transgene expression may affect endogenous *UBQ* gene expression.

To test this hypothesis, we examined the expression changes of three highly expressed endogenous *UBQ* genes, *UBQ4*, *UBQ10*, and *UBQ11*, along with the transgene *AHU*, in the same 12 d-old *6AHU BirA-6*, *-9*, and -*10* seedlings previously assayed for *BirA* expression (Figure 4B). The qPCR analysis revealed that *UBQ4*, *UBQ10*, and *UBQ11* expression declined to an average of 0.7 ± 0.1, 0.8 ± 0.1, and 0.7 ± 0.1 fold, respectively, compared to WT, when grown on DMSO-containing medium with basal *BirA* expression. Specifically, *UBQ4* and *UBQ11* were significantly downregulated in all three transgenic lines, while *UBQ10* was significantly lower in *6AHU BirA-6* and *-10* compared to the WT (Figure 4B). As expected, constitutive expression of *AHU* driven by the *UBQ10* promoter resulted in an average expression level of 6.7 ± 1.1-fold higher than the WT across the three transgenic lines.

In comparison, Dex treatment did not significantly alter this pattern. The expression of *UBQ4*, *UBQ10*, and *UBQ11* declined to an average of 0.6 ± 0.1, 0.7 ± 0.1, and 0.5 ± 0.1 fold, respectively, while *AHU* increased to an average of 8.1 ± 2.1 fold among the three transgenic lines, compared to the WT (Figure 4B). Therefore, the reduced levels of total ubiquitylated proteins are not likely attributed directly to *BirA* expression or biotinylated Ub production. However, since the three endogenous *UBQ* genes were consistently downregulated while *AHU* remained upregulated at similar levels regardless of Dex treatment, it is likely that *AHU* overexpression led to repression of endogenous *UBQ* genes, thereby reducing the overall ubiquitylation efficiency.

#### 2.4.2. Biotinylation Antagonizes Ubiquitylation by Stabilizing Ubiquitylated Proteins

Given the slight upregulation of total ubiquitylated proteins in three transgenic lines upon Dex treatment (Figure 5A), and the lack of significant impact of Dex treatment on both endogenous and transgenic *UBQ* gene expression (Figure 4B), we hypothesized that biotinylation stabilizes ubiquitylated proteins. To test this, we analyzed the proteins conjugated with *AHU*. Intriguingly, these proteins, detected by anti-6His antibodies, were dramatically increased after 16 hr of Dex treatment (Figure 5B). To determine whether these AHU conjugates were biotinylated, we probed the same protein samples using HRP-SA and observed multiple discrete bands specific to Dex-treated plants (Figure 5C). Since *BirA* expression is strongly induced by Dex through the Dex-inducible promoter (Figure 4B and Figure S1), we concluded that AHU-conjugated proteins were effectively biotinylated via the AviTag and stabilized upon induced *BirA* overexpression (Figure 5C). This stabilization effect was further supported by the time-course experiment, in which the abundance of AHU-conjugated proteins progressively increased in seedlings treated with 10 µM Dex for 0, 8, and 16 hr, but not in DMSO-treated controls (Figure 5D).

#### 2.4.3. Biotinylation Disrupts Proper Biological Functions of Ubiquitylated Proteins

Consistent with the incomplete complementation of HAT in rescuing *ask1* growth and reproductive defects, we also observed detrimental growth effects in the three *6AHU BirA* transgenic lines. This impairment appeared to be associated with *BirA* expression or the extent of biotinylation of AHU-conjugated proteins. For example, when seedlings were grown on DMSO-containing medium, *6AHU BirA-9* showed the most stunted growth and pale leaves (Figure 4A, left panel), which correlated with its highest basal *BirA* expression, 18-fold higher than in *6AHU BirA-6* and *6AHU BirA-10* (Figure 4B). However, when they were Dex-treated, significantly more biotinylated AHU-conjugated proteins were detected in the latter two lines (Figure 5B,C), which in turn led to a dramatic reduction in their seedling growth (Figure 4A, right panel).

Our previous studies have shown that ASK1-containing SCF complexes account for at least 20–30% of ubiquitylation substrates in Arabidopsis floral tissues [31]. Consequently, a large number of ubiquitylation substrates are expected to be proximally biotinylated in SCF complexes containing HAT or HALT. The negative impact of biotinylation on plant growth suggests that HAT- and HALT-mediated proximal biotinylation interferes with the normal functions of their SCF substrates in planta. Specifically, ubiquitylated proteins may fail to be efficiently degraded once biotinylated. This finding also explains the observed inverse correlation between *HAT* expression levels and its complementation efficiency, as shown in Figure 2 and Figure 3.

### 2.5. Profiling a Single Ubiquitylation Pathway Using Substrate-TurboID

The incomplete biological function of HAT and HALT, together with the antagonism between biotinylation and ubiquitylation, rendered proteome-wide SCF interactome analysis unreliable. To assess whether TurboID can still elucidate an individual ubiquitylation pathway, we focused on a single ubiquitylation substrate, the far-red-light photoreceptor phytochrome A (phyA), a well-known SCF substrate whose cognate SCF complex remains unidentified [31,33,34,35].

**Figure 6:**
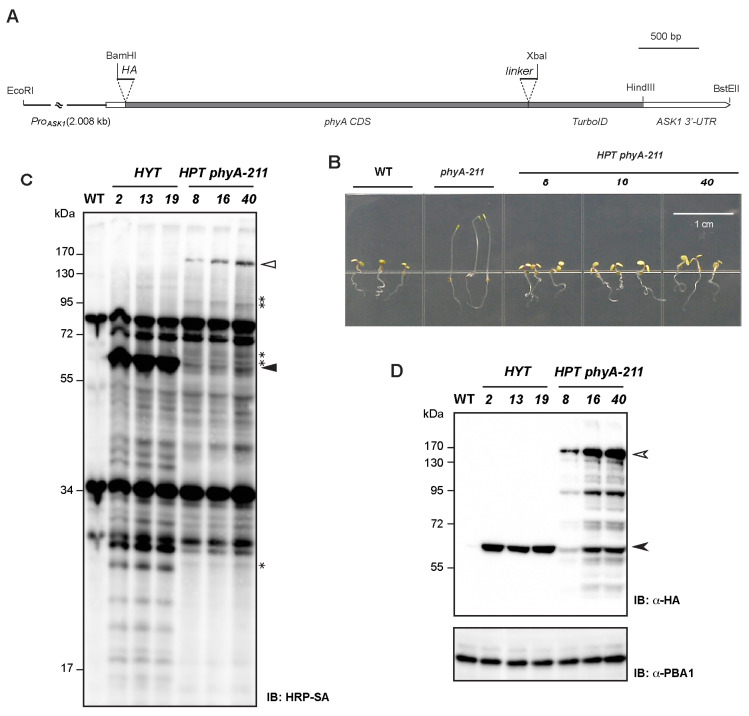
HPT with intact far-red-light response function yields multiple specific trans-biotinylation species. (**A**) Schematic diagram showing the DNA fragments and restriction sites used for construction of the *HPT* vector. (**B**) *HPT* restores the far-red-light response of the *phyA-211* mutant to WT levels in three independent transformants. (**C**) Comparison of cis- and trans-biotinylation between *HYT* and *HPT* identifies multiple HPT-specific trans-biotinylation bands (indicated with asterisks). Total protein of 4 d-old dark-grown seedlings from indicated transgenic lines and WT was used for immunoblotting analysis. Open and solid triangles indicate cis-biotinylated HPT and HYT, respectively. (**D**) Varying levels of HPT protein were detected in the three independent *HPT* transformants in the *phyA-211* background. The HYT-comparable or lower levels of HPT suggest that the HPT-specific trans-biotinylation proteins detected in (**C**) do not result from high HPT expression. Full length of HPT and HYT proteins are indicated by open and solid arrowheads, respectively. Lower-molecular-weight bands likely represent degraded HPT products. PBA1 served as a loading control, as in Figure 1C.

Using the backbone of the *HAT* constructs, we replaced the *ASK1* ORF with the *phyA* coding sequence and substituted the *ASK1* promoter with a 2-kb fragment of the *phyA* promoter (Figure 6A). The resulting *Pro_phyA_:HA-phyA-TurboID:ASK1 3′-UTR* (designated *HPT*) was transformed directly into the *phyA-211* null mutant [36].

Among 15 independent T3 homozygous lines, nine restored the far-red-light response of *phyA-211* to WT levels (Figure 6B). We selected three of them, *HPT-8*, *HPT-16*, and *HPT-40*, for basal biotinylation analysis, along with three *HYT* control lines in 4-d-old dark-grown seedlings (Figure 6C). In all three *HPT* lines, far-red-light responsiveness was fully restored despite markedly different fusion-protein abundances (Figure 6B,D). For example, the HPT fusion protein in *HPT-8* was ~10% of that in *HPT-40* (Figure 6D). Thus, basal proximal biotinylation of phyA interactors does not compromise phyA function in vivo in the far-red-light responsive pathway.

Immunoblotting analysis using HRP-SA revealed several *HPT*-specific trans-biotinylation bands that were absent from *HYT* controls (Figure 6C, asterisks), indicating robust and selective labelling of phyA-proximal proteins. Notably, the number and intensity of these bands did not strictly correlate with fusion protein abundance. For example, *HPT-8* and *HPT-40* produced comparable trans-biotinylation patterns despite a tenfold difference in their HPT fusion protein levels (Figure 6C,D). Therefore, unlike HAT, TurboID-tagged phyA labels its proximal interactors without overtly disturbing their functions.

In summary, although global biotinylation of SCF components (such as HAT and HALT) disrupts normal ubiquitylation, a substrate-centered TurboID approach may complement mutant phenotypes and yield discrete, biologically meaningful proximal interactomes. These findings demonstrate that TurboID is suitable for dissecting individual ubiquitylation pathways when the tag is confined to a single, well-behaved substrate.

## 3. Discussion

### 3.1. Antagonism Between Biotinylation and Ubiquitylation

Our results reveal a previously unappreciated antagonism between protein biotinylation and ubiquitylation. When ubiquitylated proteins are biotinylated through the AHU moieties in *6AHU BirA* transgenic plants, their turnover is retarded and the seedling growth is impaired. These findings suggest that biotinylated Ub chains are either inefficiently recognized by the 26S proteasome or actively shielded from deubiquitylation, thereby leading to proteostatic stress. Similarly, biotinylation of TurboID-fused ASK1 or CUL1, as well as their associated SCF complexes and substrates, disrupts the proper proteostasis of SCF proteomes (SCFomes), resulting in compromised biological activity. This antagonism between biotinylation and ubiquitylation explains why HAT and HALT, despite being expressed under the native *ASK1* promoter, only partially complement the *ask1* mutant (Figure 2), and why global SCF interactome mapping using TurboID-ASK1 and TurboID-CUL1^NTD^ identified only a small number of active F-box proteins [8].

### 3.2. Implications for Proximity-Labelling Studies of CRLs

Since SCF complexes are representative of the broader CRL family, similar steric or biochemical conflicts are likely to arise when PBLs are fused to other CRL subunits. Transient overexpression systems, albeit widely used in plant PL assays [8], may further exacerbate these artifacts due to the lack of complementation tests and the excessive expression from high-copy plasmid DNA delivery, unlike the stable single-copy transgenic lines analyzed here. We therefore recommend that any PL-based interactome assay of ubiquitylation pathways, including those involving plant CRLs, be interpreted cautiously unless the fusion protein is shown to rescue the corresponding loss-of-function phenotype.

Our findings further indicate that proteome-wide PL profiling of CRL pathways is unlikely to yield fully biologically relevant interactomes because biotinylation antagonizes ubiquitylation and disrupts the normal function of SCF complexes. In the case of HAT, the partial complementation of the *ask1* mutant suggests that its interactome would not faithfully reflect native SCF activity. Although short-pulse AP-MS of *HAT* lines, using *HYT* as a control, might recover a limited set of HAT-specific proximal proteins, such experiments are best suited for future exploratory and hypothesis-generating work, serving only as reference-level data rather than definitive evidence for SCF regulation.

### 3.3. A Case-by-Case Strategy: Substrate-Centered TurboID

By shifting the TurboID tag from a core SCF subunit to a single substrate (phyA), we circumvented the global antagonism of biotinylation and obtained biologically meaningful data. The HPT fusion fully restored far-red-light responses in *phyA-211*, yet produced several discrete biotinylated partners that were absent from *HYT* controls (Figure 6). Notably, trans-biotinylation signals were insensitive to a tenfold difference in HPT fusion abundance, indicating that specific labelling can be achieved effectively without perturbing interactome function. We anticipate that similar substrate-centered designs, validated by functional complementation, will be valuable for dissecting other dynamic ubiquitylation pathways (e.g., hormone receptors, signaling kinases, cell-cycle regulators, etc.).

### 3.4. Future Directions for phyA Ubiquitylation

Although several HPT-specific bands were detected, some may correspond to phytochrome-interacting factors such as PIFs, which are also ubiquitylated and degraded in a light-dependent manner [37]. Because phyA stability is differentially regulated under red versus far-red light due to rapid and Pfr-specific ubiquitylation and proteasomal degradation [38], time-resolved labelling under distinct light conditions should enrich for UPS-relevant interactors. Our HPT/phyA-211 lines therefore provide an ideal genetic platform for (i) streptavidin affinity purification and MS identification of bona fide phyA ubiquitylation factors; (ii) testing light-dependent changes in the phyA interactome; and (iii) ultimately isolating the long-sought SCF E3 ligase responsible for phyA turnover.

### 3.5. Technical Considerations and Alternative Solutions

To minimize biotinylation-induced artefacts in future PL experiments involving CRLs, several strategies could be explored, including: (i) using lower-activity PBLs (e.g., miniTurbo or UltraID) [14,15] or split-TurboID designs that reconstitute activity only upon substrate engagement [39], (ii) applying shorter labelling windows to capture transient interactions before extensive Ub biotinylation occurs, and (iii) employing orthogonal tagging approaches, such as click chemistry [40], to avoid direct modification of Ub chains. Combining these adjustments with genetic complementation assays should allow a clearer distinction between physiologically relevant interactors and biotinylation artefacts.

### 3.6. Concluding Remarks

Collectively, our study uncovers an intrinsic antagonism between biotinylation and ubiquitylation that limits the utility of TurboID for global CRL interactome profiling in plants. Nevertheless, when applied judiciously to a single, well-behaved substrate, as demonstrated for phyA, TurboID remains a powerful tool for mapping individual ubiquitylation pathways. The principles established here will inform future efforts to chart UPS networks in planta, while avoiding the pitfalls of biotin-induced proteostatic interference. While our current whole-seedling assays provide an integrated biochemical view of biotinylation–ubiquitylation interactions, future studies incorporating spatially resolved approaches (e.g., cell type–specific proximity labeling or single-cell proteomics) could reveal tissue- or cell-specific patterns that refine our understanding of these processes in planta. Additionally, because all in vitro growth conditions are inherently suboptimal and 1/2 MS medium may impose mild nutritional stress on Arabidopsis growth compared to more balanced formulations [41], it would be valuable to. examine the interplay between biotinylation and ubiquitylation under alternative growth conditions to gain further insights into their roles in plant stress responses.

## 4. Materials and Methods

### 4.1. Plant Materials and Growth Conditions

Unless otherwise noted, the Col-0 Arabidopsis reference accession was used as the WT background, and stably transformed T4 homozygous transgenic plants were analyzed. Seeds were vapor-phase surface sterilized, stratified for 3 d at 4 °C, and germinated on 1/2 MS medium containing 0.7% agar and 1% sucrose. Ten d-old seedlings were transplanted onto a soil mix containing an equal mixture of vermiculite, peat moss, and compost for propagation. Except for far-red-light assays performed under continuous far-red light or in darkness, plants were grown under LD conditions (16 h light/8 h dark; 120 µmol m^−2^ s^−1^) at 21 °C/19 °C (day/night).

### 4.2. Vector Construction and Plant Transformation

*TurboID* and *YFP* coding sequences were obtained from *TurboID-His6*_pET21a (Addgene: #107177) [14] and pEarleyGate101 (ABRC: # CD3-683), respectively. All remaining target sequences were PCR-amplified from WT cDNA or genomic DNA. The resulting PCR fragments carrying appropriate restriction sites were cloned into pCAMBIA1302 to generate *HA*-tagged *HAT*, *HYT*, and *HPT* transformation vectors.

To construct a recombinant *UBQ* gene, an *AviTag-6His* cassette was inserted at the 5′ end of the third *Ub* repeat of *UBQ11*, generating a single *AHU* unit. The resulting *AHU* moiety was cloned into NcoI-XbaI sites of pFGC5941 transformation vector [42]. Five additional *AHU* units were sequentially cloned into BamHI–XbaI sites, and a stop codon was introduced after the sixth repeat to yield the final *6AHU* transgene. A 1 kb *UBQ10* promoter was cloned as described by Hua et al. [43] and used to replace the *35S* promoter to drive *6AHU* expression. The *BirA* gene was cloned from cDNA (a gift from Dr. Richard D. Vierstra) and inserted into the XhoI-SpeI sites of pTA7002 under the control of a Dex-inducible promoter [44]. Primer sequences used in this study are listed in Table S1.

All constructs were introduced into the *Agrobacterium tumefaciens* strain GV3101, which was used to transform Col-0 for *HAT* and *6AHU*, *ASK1ask1* heterozygous plants for *HALT*, *6AHU* homozygous plants for *BirA*, and *phyA-211* for *HPT* using a floral dip method [45]. *HAT ask1* homozygous plants were obtained by crossing *HAT* homozygous plants with *ask1* mutant. *HALT ask1* homozygous plants were obtained in the progeny of *HALT ASK1ask1*.

### 4.3. Immunoblotting Analysis

Unless otherwise specified, 7-d-old LD-grown seedlings were harvested and pulverized in liquid nitrogen. For the HPT immunoblotting analysis, 4 d-old dark-grown seedlings were harvested and manually ground in liquid nitrogen under safe green light to preserve phyA. The pulverized powder was used for direct protein extraction in 2xSDS sample buffer by incubating at 95 °C for 7 min, after which the total protein extract was resolved on 10% SDS-PAGE and blotted onto an Immobilon-P polyvinylidene difluoride (PVDF) membrane (Millipore Sigma, Burlington, MA, USA). Anti-β-ACTIN, anti-6His, anti-PBA1, and anti-Ub were described as in the study of Yu and Hua [46]. HRP-SA (1:5000) and anti-HA (1:5000) were purchased from Abcam (Waltham, MA, USA) and BioLegend (San Diego, CA, USA), respectively.

### 4.4. RNA Extraction, cDNA Synthesis, and qPCR Analysis

Total RNA was extracted from 12 d-old seedlings grown under LD conditions using the NucleoSpin RNA Plus kit (Macherey-Nagel, Düren, Germany) according to the manufacturer’s protocol, followed by DNase I treatment (Thermo Fisher Scientific, Waltham, MA, USA). Three independent biological samples were prepared. The cDNA was synthesized from 5 µg of total RNA with SuperScript III (Thermo Fisher Scientific) and used as a template for qPCR with PowerUp^TM^ SYBR^TM^ Green Master Mix (Thermo Fisher Scientific), following the manufacturer’s instructions. All qPCR reactions were performed in technical triplicates on a Bio-Rad CFX Connect^TM^ Real-Time System (Bio-Rad, Hercules, CA, USA) to verify the technical consistency. For each biological replicate, the mean value of its technical triplicates was used for subsequent calculations, and only the variation among biological replicates was reported as the standard deviation (SD). Relative gene expression was calculated according to the 2^ΔΔCt^ method with *ACT2* and *PP2A* pairs as internal controls. Primer sequences used for qPCR are listed in Table S1.

### 4.5. Biotinylation Assay

For TurboID-based PL, fifty milligrams of 7 d-old LD-grown seedlings were collected and immersed in 2.5 mL of 50 μM biotin solution (Millipore Sigma). To minimize stress-induced, biologically irrelevant pathways, seedlings were incubated at room temperature with shaking at 150 rpm for short durations of 0, 15, 30, or 60 min following a 10 min vacuum infiltration, adapted from the standard TurboID labeling protocol for plants [21].

For BirA-mediated biotinylation, the same amount of 7 d-old LD-grown seedlings were immersed in 2.5 mL of 1/2 MS liquid medium containing 50 µM biotin with either 0.1% DMSO (−Dex) or 10 µM Dex (+Dex) for 16 h with shaking at 150 rpm, or for the indicated time periods in the time-course assay under LD conditions.

In both assays, seedlings were washed with ice-cold water three times to quench the biotinylation reaction before pulverization in liquid nitrogen. Total protein was extracted and assayed as described in Section 4.3.

### 4.6. Far-Red-Light Treatment

Cold-imbibed seeds were planted on 1/2 MS medium without sucrose and exposed to 120 µmol m^−2^ s^−1^ white light for 6 h to induce germination. After induction, the seeds were germinated and grown under 4.5 µmol m^−2^ s^−1^ far-red light in a custom chamber. The growth phenotype of 3 d-old seedlings was documented photographically.

## Data Availability

Data will be made available on request.

## Supplementary Materials

The following supporting information can be downloaded at: https://www.mdpi.com/article/10.3390/ijms26178248/s1.
