# Supplementary material for: Biotinylation Interferes with Protein Ubiquitylation and Turnover in Arabidopsis—A Cautionary Insight for Proximity Labeling in Ubiquitylation Proteome Studies
Source: Int J Mol Sci. 2025 Aug 25;26(17):8248. doi: 10.3390/ijms26178248 (PMC12428192; doi:10.3390/ijms26178248)
Supplement: Supplementary file 1 [file ijms-26-08248-s001.zip › ijms-3778977-supplementary.pdf]

SUPPLEMENTAL INFORMATION

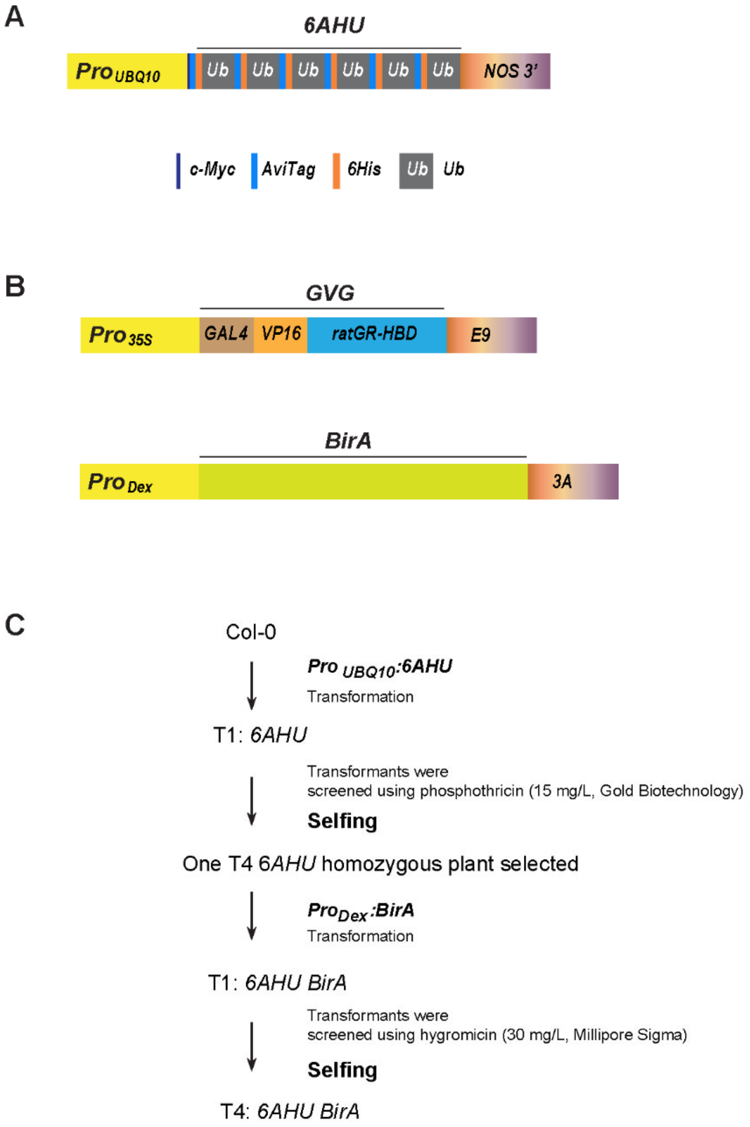

Figure S1. Developing *6AHU BirA* double transgenic plants. **(A)** A schematic diagram showing the structure of *Pro*$_{UBQ10}$:*6AHU*. *NOS 3'*: terminator of *Agrobacterium tumefaciens nopaline synthase* gene. **(B)** Structural diagrams of *GVG trans*-acting factor gene (top panel) and Dex-inducible *BirA* gene (bottom panel), adapted from [44]. Constitutive expression of GVG represses *BirA* transcription by binding to the GAL4 upstream activating sequence (UAS) within the Dex-inducible promoter. The repression is mediated by the hormone-binding domain (HBD) of the rat glucocorticoid receptor (ratGR). Upon Dex binding, repression by ratGR-HBD is relieved, enabling the strong VP16 activation domain to drive *BirA* transcription. *E9*: terminator of the pea *rbcS-E9* gene; *3A*: terminator of the pea *rbcS-3A* gene. **(C)** A flow chart showing the process of developing *6AHU* and *6AHU BirA* homozygous transgenic plants.

Table S1. List of primers used in this study.

Experiment	Name	Sequence
**Cloning**		
	ASK1_Promoter_Forward	EcoRI-ATATTTCATATTTGCATGCACG
	ASK1_Promoter_Reverse	BamHI-TTATGGAAACGAAGAGAGAAG
	HA_ASK1_8AA_Linker_Forward	BamHI-ATGTACCCATACGATGTTCCAGATTACGCTTCTGCGAAGAAGATTGTG
	HA_ASK1_8AA_Linker_Reverse	XbaI-ACCTCCGCCTCCCGCTTCAAAAGCCCATTGGTTCTC
	TurboID_Forward	XbaI-AAAGACAATACTGTGCCTCTGAAG
	TurboID_Reverse	HindIII-TCACTTTTCGGCAGACCGC
	ASK1_3UTR_Forward	HindIII-TTGATCTTCAGAGAGAAGAATC
	ASK1_3UTR_Reverse	BstEII-TTTCTTTAGCTCTTTTCGAGTGAC
	HA_YFP_8AA_Linker_Forward	BamHI-ATGGGGCGCGCCTACCCAT
	HA_YFP_8AA_Linker_Reverse	XbaI-ACCTCCGCCTCCCGCTCTAGATTACAGCTCGTCCA
	phyA_Promoter_Forward	SacI-CGTTGTCGAT TCGATCTTCTC
	phyA_Promoter_Reverse	BamHI-CCTGACACAG AGACAAGA
	HA_phyA_8AA_Linker_Forward	BamHI-ATGTACCCATACGATGTTCCAGATTACGCTTCAGGCTCTAGGCCG
	HA_phyA_8AA_Linker_Reverse	XbaI-ACCTCCGCCTCCCGCCTTGTTTGCTGCAGCGA
	21AA_Linker_TurboID_Forward	XbaI-GCGGGAGGCGGAGGTGGCGGAGGAGGCGGAGGTAGCAAGGGCGAGGAGCTGTTCAAAGACAATACTGTGCCTCTGAAG
	21AA_Linker_TurboID_Reverse	HindIII-TCACTTTTCGGCAGACCGC
	UBQ10 Promoter_Forward	EcoRI-GATCAGGATATTCTTGTTTAAGATGTTG
	UBQ10 Promoter_Reverse	NcoI-TGATCACGGTAGAGAGAATTGAG
	AHU_1st_Moiety_Forward	NcoI-ATGTCCGGCCTGAACGACATCTTCGAGGCTCAGAAAATCGAATGGCACGAAAGCAGCCATCATCATCATCATCACAGCAGCCAGATCTTCGTAAAGAC
	AHU_1st_Moiety_Reverse	XbaI-GCGGCCGCTCAGGATCCACCACGGAGACGGAG
	AHU_2nd-6th_moiety_Foward	BamHI-ATGTCCGGCCTGAACGACATCTTCGAGGCTCAGAAAATCGAATGGCACGAAAGCAGCCATCATCATCATCATCACAGCAGCCAGATCTTCGTAAAGAC
	AHU_2nd-6th_moiety_Reverse	XbaI-GCGGCCGCTCAGGATCCACCACGGAGACGGAG
	BirA_Forward	XhoI- ATGAAGGATAACACCGTGCCAC
	BirA_Reverse	SpeI- TTATTTTTCTGCACTACGCAGGG
**qPCR**		
	BirA_qPCR_F_180bp	AGTATTACGCAAGCTGGGTG
	BirA_qPCR_R_180bp	ACGACACTCTCTTCAACACG
	AHU_qPCR_F_127bp	TGACCGGAAAGACCATCACTCTTGAA
	AHU_qPCR_R_127bp	AGCTGCTTTCCAGCGAAGATG
	UBQ4_qPCR_F_71bp	GATCGCTCTTCACATCTCTTCG
	UBQ4_qPCR_R_71bp	ACCAGTGAGTGTTTTCACGA
	UBQ10_qPCR_F_117bp	TTGGAGGATGGCAGAACTCTTGCT
	UBQ10_qPCR_R_117bp	AGTTTTCCCAGTCAACGTCTTAACGAAA
	UBQ11_qPCR_F_127bp	AGCAACTTGAGGACGGCAGA
	UBQ11_qPCR_R_127bp	GTGATGGTCTTTCCGGTCAAA
